# Particularities in Surgical Results Following Obstetrical and Gynecological Surgery Using Pharmacological, Anesthesiological and Genetic Markers

**DOI:** 10.3390/jpm16020074

**Published:** 2026-01-31

**Authors:** Gabriel Valentin Tănase, Manuela Ciocoiu, Adina Elena Tănase, Ciprian Gavrila Ilea

**Affiliations:** 1 Department of Pathophysiology, Faculty of Medicine, Grigore T. Popa University of Medicine and Pharmacy, 16 University Street, 700115 Iași, Romania; gabriel-valentin.tanase@umfiasi.ro (G.V.T.); manuela.ciocoiu@umfiasi.ro (M.C.); 2 Department of Mother and Child Medicine, Faculty of Medicine, Grigore T. Popa University of Medicine and Pharmacy, 16 University Street, 700115 Iași, Romania; cilea1979@yahoo.com

**Keywords:** obstetrics, gynecology, surgery, SNP, OPRM1 gene, histopathology

## Abstract

**Aim**: Finding innovative paraclinical parameters is necessary for advancing clinical research, in obstetrics and gynecology for subjective symptoms such as pain, especially in patients with a weakened immune system, following, for example, different obstetrical and gynecological surgeries. The purpose of this study was to analyze if genetic markers can correlate with the postoperative outcome and surgical results in obstetrics and gynecology. We wanted to analyze whether patients carrying the G gene responsible for the A11G polymorphism of the OPRM1 receptor really have a higher need for analgesic doses for postoperative pain control, depending on the histopathological results, benign or malignant tumors, dimensions of tumors, type of incision performed, and hospitalization period. **Materials and Methods**: We analyzed 111 patients, including both obstetrical and gynecological cases. Blood samples (2 mL) for DNA analysis were obtained before surgery in a tube containing EDTA as an anticoagulant and immediately stored at −20 °C until required for further use. The blood samples, which were collected at the time of intravenous cannulation before surgery, were analyzed for the presence of SNP 118AG. **Results**: We examined the mutation of the opioid receptor called OPRM1 for the polymorphism noted with AG with a plus sign (+) (present) in 24.3% of the patients, with a minus sign (−) (AA) (absent) in 66.7% of the patients, and with a result with both genes modified (GG) in 9%. We correlated the data obtained in histopathology and clinical anamnesis with these results. The OPRM1(+) morphine receptor mutation was more frequently encountered in patients with biopsy uterine curettage (60%) with benign results in anatomopathology, uterine myomectomy of at least 5 cm fibromas with benign results in anatomopathology (50%), Madden mastectomy (50%), interventional hysteroscopy (33.3%) with extraction of benign tumors such as polyps or endometrial hyperplasia, caesarean section-associated surgeries (20.7%), and ovarian cystectomy (20%) (*p* = 0.048) that had a final benign anatomopathology result. **Conclusions**: Pain management in the postoperative phase is difficult for clinicians because of the response of patients to opioid therapy. Some of this variability in pain response may result from single nucleotide polymorphisms (SNPs) in the human opioid receptor mu-1 (OPRM1) that alter receptor binding or signal transduction. Part of the difficulty in identifying genes and variants that affect postsurgical pain is the inconsistent findings and poor replicability of results.

## 1. Introduction

The uterus is a vital reproductive and hormone-responsive organ that can develop a variety of pathologies, from benign to malignant. Outside of pregnancy, the uterine cavity is a virtual cavity, which undergoes changes such as endometrial hyperplasia, simple, focal, complex, or atypical; among the focal ones, the endometrial polyp is the most frequent and the cause of periovulatory bleeding. The pregnant uterus is an organ with a very important vascularization; therefore, blood loss of over 1 L is common during interventions such as transverse segmental cesarean section. If the cesarean section is associated with pathologies of the fetal appendages such as placenta previa or accreta with the penetration of placental villi into the uterine muscularis and serosa, the probability of hysterectomy and intraoperative bleeding increases exponentially [1,2,3,4,5].

The uterus is formed by the uterine corpus and the cervix; the body of the uterus is hormonally influenced and loses its endometrial mucosa once a month.

The most frequent patient visits to gynecologists are for uterine and cervical pathologies [6,7,8].

Hysterectomy, salpingo-oophorectomy, and classical or laparoscopic uterine myomectomy are the most common major gynecological surgeries in the world. They effectively treat many gynecological conditions. Several studies have described the pathological findings in these postoperative specimens and investigated the relationship between preoperative clinical diagnosis and pathological diagnosis [9,10,11,12].

The A118G polymorphism in the opioid receptor mu-1 (OPRM1) gene is associated with reduced opioid receptor availability, altered emotions, and increased acute pain after gynecological surgery. Given that emotions modulate pain (positive emotions inhibit pain, negative emotions amplify pain), we analyzed G allele carriers, who may be predisposed to experience greater postoperative pain compared to non-G allele carriers.

Endogenous opioids play a critical role in descending pain modulation and emotional processing [13,14,15,16,17].

Individual differences in opioidergic signaling may be related to altered emotional modulation of pain. The OPRM1 (opioid receptor mu-1) gene encodes mu-opioid receptors and affects emotional and pain processing. Most people have homozygous AA alleles of this receptor (i.e., individuals with the AA genotype); however, approximately 10–32% of people carry a G allele of the OPRM1 exon 1 rs1799971 polymorphism, A118G (i.e., G carriers), in which guanine replaces adenine.

It has been hypothesized that the A118G polymorphism causes a loss of function of this receptor, such that G allele carriers have impaired mu-opioid receptor functionality [18,19,20,21].

In support of this theory, G gene carriers exhibit impaired processing and management of acute postoperative pain. G gene carriers also exhibit deficits in descending pain modulation, as evidenced by the need for higher doses of opioid analgesics after surgical, gynecological, or cesarean section procedures and by greater postoperative pain, compared with AA gene carriers [22,23,24].

## 2. Materials and Methods

Gynecological and pregnant patients who presented for surgical interventions at the “Cuza Vodă” Obstetrics and Gynecology Clinical Hospital in Iași, between 1 December 2024 and 1 December 2025 and who met the following inclusion criteria and did not present any of the exclusion criteria below were part of the analysis:

The inclusion criteria were the following:

Age over 18 years;

Associated obstetric or gynecological surgery;

ASA physical status (according to the American Society of Anesthesiologists) II or III.

Exclusion criteria included the following:

Patients under 18 years of age;

Persons with a body mass index greater than 40 kg/m^2^, with suspicion of sepsis or serious medical pathology and associated severe comorbidities, psychiatric or cardiopulmonary disorders, decompensated diabetes mellitus and patients who chronically use analgesics.

All patients signed the consent form and had the purpose of this study explained to them.

A limitation of this study is the variability in analgesic prescribing practices that can substantially influence pain outcomes independently of genetic factors. However, we followed the same postoperative protocol of medications usually prescribed for pain management in our hospital.

## 3. Statistical Data

Statistical processing included data analysis in SPSS 18.0 and processing by statistical functions up to the 95% significance level. Both descriptive and analytical methods were used. After collecting the data, in an accessible form to ensure their informational nature, their processing was carried out. The processing actions included the stage of ordering, systematization, centralization and reduction of the volume of information collected, through successive operations of grouping, sorting, coding and synthesizing.

The data were expressed in a form that allows them to be classified into certain categories; a variable will not be recorded in more than one form; data grouping will be performed by variable categories. The data were centralized in EXCEL and SPSS databases and processed with the statistical functions to which they lend themselves.

In the presentation of the data, confidence intervals at the 95% significance level were used, and for testing the differences, the χ2 and *t*-Student tests were used.

Primary processing, the respective systematization of the data gathering, led to obtaining the primary indicators, which are presented in the form of absolute values.

Derived indicators have the role of highlighting the qualitative aspects of an ensemble, targeting the relationship between different parts of a group of patients or different characteristics and interdependent links between variables. The following derived indicators were used:

- Indicators of the average value: simple arithmetic mean, median, modulus;
- Indicators of dispersion: standard deviation, variation.

Testing the normality of the quantitative value series was performed with the Kolmogorov–Smirnov test used to test large data sets. The threshold chosen in testing the null hypothesis is *p* > 0.05; therefore, the variables are normally distributed.

A total of 111 patients were included in our study. This study included all types of obstetrical and gynecological surgeries such as caesarean section, hysterectomies such as vaginal and total abdominal hysterectomies, and unilateral/bilateral salpingectomy/salpingo-oophorectomy, myomectomy and breast surgery—Madden radical mastectomy, or small surgeries such as biopsy uterine curettage or interventional hysteroscopy for endometrial polyps or endometrial hyperplasia. Hysterectomy for gynecological cancer and pregnancy complications was not considered. The information was gathered from the patients’ theater records, histopathology reports, and case notes and entered into a predesigned proforma. The data set included socio-demographic characteristics, clinical presentation, type of hysterectomy, clinical diagnosis, and pathological lesions.

### 3.1. Laboratory Analyses

Blood samples, which were collected at the time of intravenous cannulation before surgery, were analyzed for the presence of SNP 118AG using the following technique: Genomic DNA was extracted from 3 mL of venous blood using the genetic blood kit. The DNA was qualitatively and quantitatively verified using NanoDrop. Genotyping for the A118G polymorphism (rs1799971) was performed using the Taqman SNP genotyping assay.

### 3.2. Amplification

Amplification was performed in a 12 μL volume containing 25 ng of genomic DNA, Taqman Universal. The PCR master mix contained 60 nM of each probe and 270 nM of each primer.

Cycling and hybridization conditions were set according to the manufacturer’s instructions. The 50 cycles of denaturation and annealing/extension and quantification of fluorescent intensity by post-polymerase chain reaction were performed using the Applied Biosystems 7300 Real-Time PCR System.

## 4. Results

**Figure 1:**
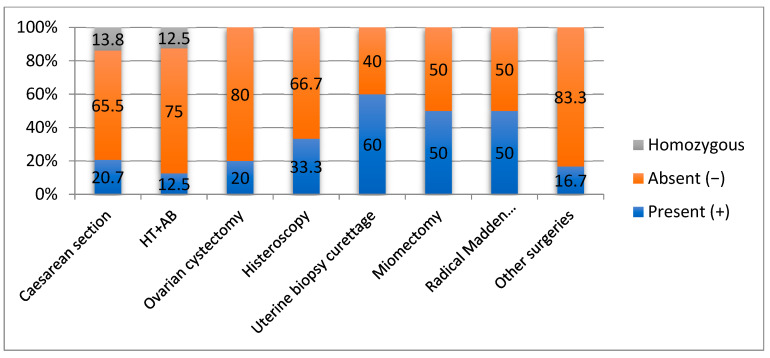
Correlation of OPRM1 gene mutations with surgical intervention.

This study included all types of obstetrical and gynecological surgeries such as caesarean section, hysterectomies such as vaginal and total abdominal hysterectomies, and unilateral/bilateral salpingectomy/salpingo-oophorectomy, myomectomy and breast surgery—Madden radical mastectomy, or small surgeries such as biopsy uterine curettage or interventional hysteroscopy for endometrial polyps or endometrial hyperplasia. For the total 111 surgeries, the most common gynecological surgery was hysterectomy, and the most common type of hysterectomy was total abdominal hysterectomy (84.6%). The most common clinical indications for hysterectomy were leiomyoma (66.8%), followed by adenomyosis (5.4%). The most common findings identified by histopathology were proliferative endometrium (38.6%) in the endometrium, leiomyoma (66.8%) in the myometrium, chronic cervicitis (3.5%) in the cervix, and endometriosis cysts (40.6%) in the ovaries (Figure 1). Histopathological confirmation of preoperative diagnosis was 88.6% for cervical pathology.

The main evaluation endpoint was correlation between opioid and non-opioid also known as analgesic medication and correlation with positive OPRM1 polymorphism.

Each patient signed a consent form, approved by the ethical committee. The majority of the cases preoperatively diagnosed as menometrorrhagia were found to have adenomyosis, endometrial polyp, secretory endometrium, or disordered proliferative endometrium.

We examined the mutation of the opioid receptor called OPRM1 for the polymorphism noted with AG with a plus sign (+) (present) in 24.3% of the patients, with a minus sign (−) (AA) (absent) in 66.7% of the patients, and with a result with both genes modified (GG) in 9%.

### 4.1. Demographic Characteristics of the Patients Analyzed

The age of the patients with OPRM1(+) ranged from 20 to 79 years, recording a mean level of 40.48 ± 15.15, while in the patients with OPRM1(−) the age ranged from 23 to 69 years, recording a mean level of 36.38 ± 9.75 (*p* = 0.331). By age groups: 51.9% of the patients with OPRM1(+) and 51.4% of those with OPRM1(−) were over 36 years old (*p* = 0.965).

**Figure 2:**
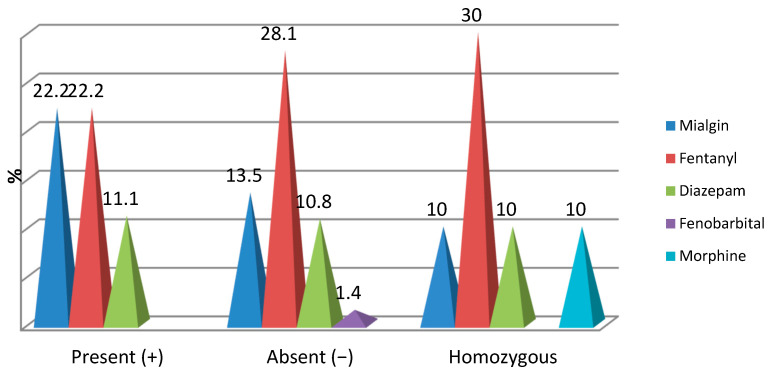
Correlation of OPRM1 gene mutations with opioid medication.

By origin: 70.4% of patients with OPRM1(+) and 67.6% of those with OPRM1(−) came from urban areas (*p* = 0.790) (Figure 2, Table 1). Of the 10 patients with both modified genes, 60% were under 36 years of age (*p* = 0.629) and 80% came from urban areas (*p* = 0.770).

### 4.2. Clinical Data Analyzed

For the obstetrical group, pregnancy in patients with OPRM1(+) ranged from 1 to 3 pregnancies, on average approximately 2, and in patients with OPRM1(−) ranged from 1 to 4 pregnancies, on average approximately 2 (*p* = 0.960).

Parity in patients with OPRM1(+) ranged from 1 to 3 births, with an average of approximately 2, and in patients with OPRM1(−) ranged from 1 to 4 births, with an average of approximately 2 (*p* = 0.918).

Gestational age in patients with OPRM1(+) ranged from 37 to 40 weeks, with an average of 38.08 ± 1.17, similar to patients with OPRM1(−), where gestational age ranged from 31 to 40 weeks, with an average of 38.00 ± 1.56 (*p* = 0.999).

The frequency of patients with a gestational age of 31–37 weeks was reduced in both groups (18.5% vs. 6.8%; *p* = 0.025) (Table 1). Of the 10 patients with both modified genes, 80% had 1–2 pregnancies and 60% had 1–2 births and 50% had a gestational age of 31–37 weeks.

### 4.3. Associated Medical Pathology

In the gynecology group, approximately 1/3 of patients with OPRM1(+) and those with OPRM1(−) showed the presence of iron deficiency anemia (29.6% vs. 29.7%; *p* = 0.992). Adherence syndrome was slightly more frequent in patients with OPRM1(−) (11.1% vs. 13.5%; *p* = 0.751). The other associated pathologies did not show statistically significant percentage differences (*p* > 0.05).

### 4.4. Length of Stay in Hospital

#### Surgical Procedures and Anatomopathology Results Performed in This Study

The OPRM1(+) morphine receptor mutation was more frequently present in patients with biopsy uterine curettage (60%) with benign results in anatomopathology, uterine myomectomy of at least 5 cm fibromas with benign results in anatomopathology (50%), Madden mastectomy (50%)—anatomopathology stadialization was performed, interventional hysteroscopy (33.3%) with extraction of benign tumors, caesarean section (20.7%), and ovarian cystectomy (20%) (*p* = 0.048) that had a final benign anatomopathology result.

Duration of hospitalization: The average number of days of hospitalization was highest in patients with OPRM1(+), and lowest in patients with both modified genes (6.32 ± 4.39 vs. 4.70 ± 1.34; *p* = 0.592).

Days of acute pain during hospitalization: The average number of days of acute pain during hospitalization was approximately 3 regardless of the results of OPRM1 gene mutations (3.33 vs. 3.19 and 3, respectively; *p* = 0.811).

Intraoperative associated interventions: Among patients with OPRM1(+), 14.8% had intraoperative small piece extraction (*p* = 0.060) and 11.1% drainage (*p* = 0.648). Among patients with OPRM1(−), 14.9% had intraoperative adhesiolysis (*p* = 0.238). Among patients with both altered genes, 30% had intraoperative adhesiolysis (*p* = 0.238) and 20% drainage (*p* = 0.648).

### 4.5. Intraoperative Drug Treatment

Ibuprofen, one dose, was found in the intraoperative treatment in all patients with OPRM1(+) and patients with both modified genes, as well as in 73.7% of patients with OPRM1(−) (*p* = 0.616).

Paracetamol, one dose, was found most frequently in intraoperative treatment in 50% of patients with OPRM1(+) and 44.4% of patients with both modified genes, as well as in 34.9% of patients with OPRM1(−) (*p* = 0.071). Mabron, one dose, was found most frequently in intraoperative treatment in 64.7% of patients with OPRM1(+), 61.2% of patients with OPRM1(−) and 60% of patients with both modified genes (*p* = 0.817). Algocamine, two doses, was found most frequently in intraoperative treatment in 44.4% of patients with OPRM1(+) and 47.5% of patients with OPRM1(−), and in patients with both modified genes, 55.6% had intraoperative treatment with one dose of Algocamine (*p* = 0.355). Acupan, one dose, was found in the intraoperative treatment in 92.9% of patients with OPRM1(+), in 66.7% of patients with OPRM1(−), and in 75.0% of patients with both modified genes (*p* = 0.263). Refen, one dose, was found most frequently in the intraoperative treatment in 71.4% of patients with OPRM1(+), 54.4% of patients with OPRM1(−), and 44.4% of patients with both modified genes (*p* = 0.110).

Intraoperatively, the distribution of cases with opioid medication was found with low frequencies and did not register significant percentage differences depending on the mutations of the OPRM1 gene:

- Mialgin was found intraoperatively in 22.2% of patients with OPRM1(+), 13.5% of patients with OPRM1(−) and 10% of patients with both modified genes (*p* = 0.515);
- Fentanyl was identified intraoperatively in 22.2% of patients with OPRM1(+), 28.1% of patients with OPRM1 (−) and 30% of patients with both modified genes (*p* = 0.802);
- Diazepam was identified intraoperatively in 11.1% of patients with OPRM1(+), 10.8% of patients with OPRM1 (−) and 10% of patients with both modified genes (*p* = 0.995);
- Phenobarbital was identified intraoperatively in only 1.4% of patients with OPRM1(−) (*p* = 0.665);
- Morphine was administered intraoperatively in only 10% of patients with both modified genes (*p* = 0.086) (Figure 2).

### 4.6. Postoperative Medication Used

Clexane was found in the postoperative treatment of 70.4% of patients with OPRM1(+), 75.7% of patients with OPRM1(−) and all patients with both modified genes (*p* = 0.052). Cefotax was identified in the postoperative treatment only in 22.2% of patients with OPRM1(+), 16.2% of patients with OPRM1(−) and 10% of patients with both modified genes (*p* = 0.634). Refen was identified in the postoperative treatment in 90.5% of patients with OPRM1(+), 70.3% of patients with OPRM1(−) and 90% of patients with both modified genes (*p* = 0.634). Patients treated with Algocamin intraoperatively, depending on the result of OPRM1 gene mutations, had the following percentage distribution: 71.4% of patients with OPRM1(+), 64.8% of patients with OPRM1(−) and 80% of patients with both modified genes (*p* = 0.576).

Post-treatment analgesic evolution: Post treatment, the patients’ condition improved in 66.7% of patients with OPRM1(+), 74.3% of patients with OPRM1(−) and 70% of patients with both modified genes (*p* = 0.666).

## 5. Discussion

Opioid analgesics are used worldwide for the treatment of moderate to severe acute and chronic pain in general hospitals. However, the occurrence of opioid-related side effects, such as respiratory depression, nausea, vomiting, constipation, and sedation, can limit dosing and affect the efficacy of opioid treatment [18,19,20].

This can lead to poor patient compliance, discontinuation of therapy, underdosing of drugs, and inadequate analgesia. Conversely, prolonged use of opioids, as in the treatment of chronic pain, can also lead to tolerance and adverse effects, such as hyperalgesia and dependence, which may limit their efficacy. Among the genes involved in opioid pharmacodynamics, the opioid receptor mu-1 (OPRM1) gene has been investigated in various pharmacogenetic studies. OPRM1 encodes the μ opioid receptor, which is the main target of both endogenous and clinically relevant opioids, such as morphine and fentanyl [21,22].

The μ-opioid receptor belongs to the rhodopsin family of G protein-coupled receptors (GPCRs) and consists of an extracellular N-terminus, seven transmembrane helices, three extracellular and intracellular loops, and an intracellular C-terminus. Human OPRM1 is located on chromosome 6q24-q25 and spans over 200 Kb, with at least nine exons and 19 different splice variants under the control of multiple promoters, and contains over a hundred SNPs. In particular, 118A>G (database SNP [dbSNP] accession no. rs1799971) is the most studied variant in pharmacogenetic research on opioid drugs [23,24].

This SNP is located in exon 1 of the gene and consists of an adenine (A) to guanine (G) substitution, which in turn causes an amino acid change at position 40 of the μ-opioid receptor protein from asparagine to aspartic acid (N40D), resulting in the loss of an N-glycosylation site in the extracellular region of the receptor. The variant allele (118 G) has a frequency of 27–48% in Asians, 11–17% in Caucasians, 2.2% in African Americans, and 0.8% in sub-Saharan Africans (dbSNP Short Genetic Variations database of the US National Center for Biotechnology Information, NCBI, Bethesda, MD, USA, accessed 1 December 2012); therefore, it is carried frequently enough to be of clinical interest for opioid therapy [20,22].

The power in lipid solubility of these drugs influences their analgesic effects. Therefore, variations in pain perception or analgesic response to a painful procedure may be caused by single nucleotide polymorphisms (SNPs) in the DNA molecule encoding a protein involved in the pain pathway.

It is estimated that genetic polymorphisms can explain up to 30% of the variability in opioid dosage requirements. The 118A > G SNP (rs1799971) in the opioid receptor mu-1 (OPRM1) gene is one of the most frequently investigated SNPs for modulating opioid analgesic response, with the G allele being associated with less effective pain relief with standard doses of opioid analgesics. This OPRM1 receptor polymorphism may alter MOP signals and influence the neurons, which affect the main functions of the opioid receptor [17,18,19].

This polymorphism is being intensively studied in all surgical branches including pediatric orthopedics [13], where it seems that the OPRM1 polymorphism could explain different responses in the postoperative pain perception of patients over 12 years old and could be a tool for proper doses of analgesics.

A higher presence of AG heterozygotes was also found in our study, the normal or wild variant GG also being found, and the homozygous AA with both mutant genes being rarer in this study group.

This polymorphism has also been studied in cardiac surgery (Matic et al. 2020) [14]. Investigation of the potential role of OPRM1 (opioid receptor mu-1) and COMT (catechol-O-methyltransferase enzyme) polymorphisms have been performed in cases of acute, chronic and experimental postoperative thermal pain. The COMT haplotype seems to explain part of the variability in acute postoperative pain in adult patients undergoing cardiac surgery.

A team from Italy investigated this polymorphism in pregnant women: a cohort of 63 parturient women, scheduled for elective cesarean section at a tertiary university hospital, received spinal anesthesia with hyperbaric bupivacaine and morphine 100 mcg. In the first 48 h of the postoperative period, the patients received acetaminophen 1 g IV every 6 h. Incident pain was treated with ketorolac 30 mg IV. The following parameters were recorded every 6 h: Visual Analogue Scale (VAS) at rest, VAS during movement, postoperative nausea and vomiting (PONV), pruritus, need for rescue analgesic medication. The results showed that of the 63 enrolled patients, 45 (71%) were homozygous genotype A/A (group 118A), while 18 were carriers of the G variants of the OPRM1 gene (A/G or G/G) (group 118G). No significant differences were observed in the administration of rescue analgesic doses and in the incidence of moderate/severe postoperative pain (VAS > 3) between the two groups. Pruritus was more frequent in group 118A than in group 118G in the first 24 h of the postoperative period. Thus, in the Italian population there is a different incidence of pruritus in the post-cesarean period in response to intrathecal opioids related to the OPRM1 gene polymorphism, but not to postoperative pain [15].

Several studies have attempted to identify genetic determinants of the clinical response to opioids administered during labor or after cesarean section. However, their results are often contradictory.

Giacon et al. (2025) [1] investigated this polymorphism in labor. The authors showed that several polymorphisms may be involved in the management and treatment of acute pain. Twenty-six studies enrolling 7765 patients were included in the systematic analysis. Overall, they analyzed several included studies, but considered a total of 12 candidate polymorphic genes (OPRM1, COMT, CYP2D6, CYP3A4, ABCB1, ABCC3, UGT2B7, CGRP, OPRK1, OPRD1, KCNJ6, KCNJ9), of which the most investigated variant was OPRM1 rs1799971. The overall pooled results indicated that individuals carrying the G allele of the OPRM1 rs1799971 gene required higher doses of opioids for pain management compared with subjects with the AA rs1799971 allele (standardized mean difference: 0.26; 95% CI: 0.09–0.44; *p* = 0.003). Such an association was confirmed in the subgroups of patients with labor pain and post-cesarean section pain. This information provides strong evidence of an association between OPRM1 rs1799971 and the required dose of opioids for labor or post-cesarean pain relief. Larger studies are needed to investigate the impact of genetic variability on the efficacy and safety of opioid medications for labor and post-cesarean pain relief.

Landau et al. (2013) [12] investigated intravenous fentanyl used as an analgesic in labor, according to the combined effect of single nucleotide polymorphisms rs1799971 (c.118A/G, p.40Asn/Asp) of the opioid receptor mu-1 (OPRM1) gene and rs4680 (c.472G/A, p.158Val/Met) of the catechol-O-methyltransferase (COMT) gene in women requesting analgesia in labor. One hundred and six women were enrolled and received intravenous fentanyl. Intravenous analgesic success was 6% in women with the Asn/Asn-Met/Met combination (n = 17) versus 20% in all other women combined. Intravenous analgesic success was 20% in women with 118A/A (Asn/Asn) versus 21% for A/G and G/G in OPRM1 and 10% in women with 472A (Met/Met) versus 22% for A/G (Met/Val) and G/G (Val/Val) in COMT.

Primary dysmenorrhea (PDM) is a common condition among women of reproductive age admitted to the gynecological department, characterized by menstrual pain in the absence of any organic causes. Previous research has established that carriers of the G allele exhibit maladaptive functional connectivity between the descending pain modulatory system and the motor system in young women with PDM.

A study conducted by Hsu et al. (2023) [2] explored the potential relationship between the OPRM1 A118G polymorphism and white matter changes in young women with primary menstrual dysmenorrhea individuals with primary dysmenorrhea, including 13 AA homozygotes and 30 G allele carriers. Diffusion tensor imaging (DTI) scans were performed during both the menstrual and periovulatory phases, and tract-based spatial statistics (TBSS) and probabilistic tractography were used to explore white matter microstructure variations related to the OPRM1 A118G polymorphism. The short-form McGill Pain Questionnaire (MPQ) was used to assess participants’ pain experience during the MEN phase. The OPRM1 A118G polymorphism was found that it may influence the association between structural integrity and dysmenorrheal pain, where the G allele could impede the pain-regulating effects of the A allele.

Given that this study took place in the largest hospital in the northeastern region of Romania, its limitations and the variable number of patients analyzed are associated with the diversity of gynecological surgical interventions performed; the extemporaneous results obtained in real time, which changed the diagnosis from benign to oncological; and the diversity of indications for cesarean section.

## 6. Conclusions

Postoperative pain treatment is a permanent challenge for medical healthcare workers because of the unpredictable responses of patients to opioid treatment.

Some of this variability in pain response may result from single nucleotide polymorphisms (SNPs) in the human opioid receptor mu-1 (OPRM1) that alter receptor binding or signal transduction.

Part of the difficulty in identifying genes and variants that affect postsurgical pain is the inconsistent findings and poor replicability of results.

Postsurgical pain is assessed in some studies by opioid consumption, in some studies by patient-reported pain scores, and in some studies by both measures. Most studies have assessed pain either in the acute period (0 to 48 h postoperatively) or in the chronic period (at least 3 months postoperatively); therefore, more universally standardized questionnaires are needed for accurate quantification.

Each person’s different reactions to fentanyl, for instance—a frequent opioid analgesic—and SNP may interact with effective pain treatment doses. The opioid addiction epidemic is emerging as one of the most serious clinical problems of our time. Opioids are highly addictive after first exposure.

Molecular, genetic, epigenetic, and environmental variations are also implicated in the development of opioid addiction.

Patients with higher levels of pain after surgery are more likely to have persistent pain and greater opioid use, contributing to a global epidemic of chronic pain that has an annual cost to society greater than that of cancer, heart disease, and diabetes, the world’s leading medical conditions.

The results obtained from the present research on genes and variants associated with postsurgical pain can be explained in part by the different patient and surgical populations analyzed in the literature to date.

The OPRM1 A118G allele (rs1799971) is the most studied variant in relation to postsurgical pain, with several studies reporting increased pain scores and opioid requirements with the G allele; therefore, the data published in our article should be considered when approaching this pathology in the obstetrical and gynecological intensive care unit departments. However, these conclusions are preliminary important observations that will allow support for further research in order to improve the scientific balance of opioid management.

## Figures and Tables

**Table 1:** Associated pathology according to polymorphism.

Pathology	OPRM1(+) (*n* = 27)		OPRM1(−) (*n* = 74)		Chi2 Test *p*
	*n*	%	*n*	%	
Iron deficiency anemia	8	29.6	22	29.7	0.992
Uterine fibroid	1	3.7	6	8.1	0.443
Cord ring	1	3.7	3	4.1	0.937
Thrombophilia	1	3.7	6	8.1	0.443
Arterial hypertension	2	7.4	1	1.4	0.114
Diabetes mellitus	2	7.4	1	1.4	0.114
IVF	1	3.7	2	2.7	0.794
Adherence syndrome	3	11.1	10	13.5	0.751

## Data Availability

The original contributions presented in this study are included in the article. Further inquiries can be directed to the corresponding author.
